# The Impact of Dabigatran and Rivaroxaban on Variation of Platelet Activation Biomarkers and DRT Following Percutaneous Left Atrial Appendage Closure

**DOI:** 10.3389/fphar.2021.723905

**Published:** 2021-09-15

**Authors:** Xiaoye Li, Xiaochun Zhang, Qinchun Jin, Yanli Li, Daxin Zhou, Qianzhou Lv, Junbo Ge

**Affiliations:** ^1^ Department of Pharmacy, Zhongshan Hospital, Fudan University, Shanghai, China; ^2^ Department of Cardiology, Zhongshan Hospital, Fudan University, Shanghai, China

**Keywords:** direct oral anticoagulants, percutaneous left atrial appendage closure, device-related thrombosis, thrombin receptor–activating peptide–induced platelet aggregation, platelet activation biomarkers, thrombin–antithrombin complex, P-selectin, von Willebrand disease

## Abstract

**Background**: The current post-procedure antithrombotic recommendation for left atrial appendage closure (LAAC) remains empiric. This study was designed to compare variations in platelet activation biomarkers and device-related thrombosis (DRT) under different antithrombotic regimens following LAAC.

**Methods:** This study enrolled 105 consecutive patients with atrial fibrillation who underwent LAAC successfully and received post-procedure anticoagulation with either dabigatran (*N* = 33) or rivaroxaban (*N* = 72). After 3 months of anticoagulation treatment, thromboelastogram was used to evaluate thrombin receptor–activating peptide (TRAP)–induced platelet aggregation (PA). Measurements of platelet activation biomarkers, including thrombin–antithrombin complex (TAT), P-selectin, von Willebrand disease (vWF), and CD40L, were performed immediately before the LAAC procedure and after 3 months of post-procedure anticoagulation. Repeated transesophageal echocardiography was performed to evaluate DRT during follow-ups.

**Results:** Three (4.2%) patients in the rivaroxaban and 4 (12.1%) patients in the dabigatran group experienced DRT events (odds ratio (OR) = 0.315, 95% confidence interval (95%CI): 0.066–1.489, *p* = 0.129) during follow-ups. The TRAP-induced PA was statistically significantly higher in the dabigatran group (62.9% vs 59.7%, *p* = 0.028^*^). Statistically significant increases in plasma concentration of TAT, P-selectin, and vWF were observed after 3 months of exposure to dabigatran when compared with rivaroxaban. An increased expression of platelet activation biomarkers was observed in DRT subjects compared with non–DRT subjects in terms of P-selectin and vWF (65.28 ± 13.93 ng/L vs 32.14 ± 12.11 ng/L, *p* = 0.037; 501.92 ± 106.48 U/L vs 280.98 ± 54.10 U/L, *p* = 0.045; respectively). Multivariate regression analysis indicated that the use of dabigatran might be an independent predictor of DRT (*p* = 0.022; OR = 4.366, 95%CI: 0.434–10.839). Furthermore, the CHA_2_DS_2_-VASc score (OR = 2.076, *p* = 0.016) and CD40L levels (OR = 1.015, *p* = 0.021) were independent predictors of increased D-dimer levels.

**Conclusions:** Post-LAAC anticoagulation with dabigatran may increase the risk of DRT by enhancing platelet reactivity. In light of this potential increased risk in DRT, the authors recommend against using dabigatran for post-procedural anticoagulation in patients who have undergone LAAC.

## Introduction

Percutaneous left atrial appendage closure (LAAC) has become an effective and safe surgical method for the prevention of stroke. It is mainly available for patients who are diagnosed with non–valvular atrial fibrillation (NVAF) and who cannot adhere to long-term anticoagulant therapy (Iskandar et al., 2016; Reddy et al., 2017; Reddy, 2018). It is widely known that thrombosis in the left atrium can increase the risk of stroke and about 90% of identified left atrial thrombosis are located in the left atrial appendage (LAA) (Saw et al., 2019). Therefore, LAAC has been used to potentially reduce the occurrence of thrombosis and bleeding events in patients with NVAF (Reddy et al., 2013; Boersma et al., 2017).

Similar to other implanted devices in the human body, when the occluders used for LAAC are exposed to the circulating blood, it takes time for complete endothelialization of the occluders. Meanwhile, thrombosis may occur to the exposed device, therefore adequate antithrombotic treatment is required to prevent device-related thrombosis (DRT) (Fauchier et al., 2018; Asmarats et al., 2019; Duthoit et al., 2020). However, there is a remarkable interindividual variability on the time taken for full endothelialization, which causes uncertainty on the duration of anticoagulation in patients with LAAC (Gouin-Thibault et al., 2017). Current guidelines recommend that patients could be on direct oral anticoagulation (DOAC) after LAAC operation for 3 months to prevent DRT, then dual antiplatelet therapy should be continued for up to 6 months, followed by taking aspirin lifelong when DRT is excluded (Glikson et al., 2019). Previous clinical trials have shown superior effectiveness and safety with using DOAC agents than with antiplatelet agents in terms of lowering DRT occurrence after LAAC (Bergmann et al., 2017).

Recently, clinical trials have prompted a warning against using dabigatran as the anticoagulant in patients with mechanical heart valves mainly because of the enhancement of platelet aggregation (PA) (Eikelboom et al., 2013), while rivaroxaban, a selective Xa inhibitor, has been confirmed to decrease clot formation induced by thrombin and has remained favorable for patients with vascular thrombosis in the COMPASS trial (Sharma et al., 2019). Case reports and retrospective trials have reported DRT when using dabigatran for post-procedure anticoagulation following LAAC (Li et al., 2020). However, the mechanism for dabigatran-associated DRT remains unclear. It is well known that enhanced platelet activity might be an important independent risk factor for thrombosis formation and platelet activation, and biomarkers such as thrombin–antithrombin complex (TAT), P-selectin, von Willebrand disease (vWF), and CD40L could be used to reflect platelet activity (Ceriello et al., 2014). Therefore, the objective of this study was to evaluate platelet reactivity with different antithrombotic regimens following occluder implantation.

## Methods

### Study Design and Population

This is a single-center, observational study. Patients who received dabigatran or rivaroxaban post-LAAC in the Department of Cardiology, Zhongshan Hospital, Fudan University, between January 2018 and December 2019 were enrolled. All patients undergoing LAAC present with a high risk for stroke, transient ischemic attack, and systemic embolism with a CHA_2_DS_2_-VASc score ≥2 and are deemed to be poor candidates for long-term oral anticoagulation (OAC). The main exclusion criteria were as follows:1) concomitant anti-platelet medication, 2) severe renal dysfunction, 3) severe hepatic insufficiency, and 4) discontinuation of anticoagulation with the use of dabigatran or rivaroxaban. Outpatient follow-up visits on transesophageal echocardiography (TEE) were scheduled at 3 months after LAAC. Platelet function and platelet activation biomarkers were measured. Adequate venous blood samples were collected to evaluate the platelet activation biomarker variations at baseline right before the procedure and after 3 months of anticoagulation. Demographic and baseline characteristics through the electronic medical record system were obtained. According to the European Society of Cardiology (ESC) criteria, NVAF was diagnosed using electrocardiogram (ECG) showing a typical pattern of atrial fibrillation—absolutely irregular RR intervals and no discernible and distinct P waves (Kirchhof et al., 2016). The Medical Ethics Committee of Zhongshan Hospital approved this study and waived the requirement for informed consent. This study was well performed in compliance with the principles of the Declaration of Helsinki.

### Direct Oral Anticoagulation Agents Administration

All patients were administrated with DOAC agents before LAAC procedure. The choice of DOAC agent was mainly dependent on clinician preference or evidence-based medicine. Dabigatran and rivaroxaban were the DOAC agents available at the hospital where this study was carried out. Based on the anticoagulation agents, patients were categorized into the dabigatran (110 mg b.i.d., the only available dose) group and rivaroxaban (20 mg q.d.) group. Dosages were used according to the drug package inserts. The decision to initiate anticoagulation following LAAC operation was made on a case-by-case basis considering the individual's risk of bleeding and renal function, at the discretion of the cardiologist. Dabigatran was chosen on the basis of the consideration factors, such as high bleeding risk and renal function with eGFR >30 ml/min·per 1.73 m^2^. The DOAC agents were discontinued and heparin was used for bridging on the day of the operation. After the operation, all patients were continuously medicated with a 3-month course of DOAC to facilitate device endothelialization followed by dual antiplatelet therapy until 6 months and then lifelong aspirin use.

### Left Atrial Appendage Closure Operation Procedure

Briefly, the closures were implanted under conditions of general anesthesia and fluoroscopic guidance *via* the femoral vein and transseptal access. Based on these criteria, percutaneous LAAC was performed using the occluders (WATCHMAN, Boston Scientific, Natick, MA, United States) by referring to marker bands (21, 24, 27, 31, and 33 mm). Intraprocedural TEE was applied to rule out LAA thrombosis and define the LAA dimensions for device sizing. The operation was conducted in a routine manner, and all patients obtained successful device implantation.

### Platelet Aggregation Measurement

Thromboelastogram (TEG) was applied to analyze PA stimulated by thrombin receptor–activating peptide (TRAP, 32 μM) after 3-month anticoagulation. The mechanical electrical transducer was applied to monitor the coagulation status of the whole blood, and changes of amplitude were recorded during thrombosis. The maximum amplitude (MA) was defined as the largest change of coagulation intensity amplitude value. The tested MA was classified into thrombin-induced MA (MA_thrombin_), TRAP-induced MA (MA_TRAP_), and fibrin-induced MA (MA_Fibrin_), depending on which of the three different activators was added to the blood sample. The TRAP-induced PA was calculated by the formula: TRAP-induced PA (%) = (MA_thrombin_ − MA_TRAP_)/(MA_thrombin_ − MA_Fibrin_).

### Laboratory Parameters

A venous blood sample (2 ml) was collected from each patient at the baseline right before the procedure and 3 months post-anticoagulation after LAAC operation under standard conditions and stored at −80°C for further analysis. In addition to the standard coagulation markers (activated partial thromboplastin time, prothrombin time, and international normalized ration (INR)), platelet activation biomarkers those which reflect platelet activity were measured. The enzyme-linked immunosorbent assay kit recommended in the manufacturer’s instructions was used to measure platelet activation biomarkers, including TAT, P-selectin, vWF, and CD40L with detection ranges of 2–100 ng/ml, 10–1,000 ng/L, 20–500 U/L, and 30–2,400 ng/L, respectively. Plasma D-dimer levels were detected by immunoturbidimetry using ACL TOP 700 system (Beckman Coulter Inc. Fullerton, CA, United States) according to the manufacturer’s instructions. Concentration of D-dimer < 0.5 mg/L was regarded as the normal level. All these tests were performed according to the standard operating procedures of the instrument.

### Device-Related Thrombosis

Similar to other implanted devices in the human body, a thin layer of fibrin may form on the device due to exposure to blood circulation after LAAC. DRT was defined as a well-circumscribed echo-reflective mass on the left atrial (LA) side of the device, and the size of thrombosis was assessed by TEE. As for the DRT appearance, anticoagulants might be switched from DOAC agents to warfarin within therapeutic the INR.

### Data Collection

Detailed demographic and baseline information of each subject including history of smoking or alcohol consumption, comorbidity disease, levels of hemoglobin (Hb) and hematocrit (Hct), platelet (PLT) count, alanine aminotransferase (ALT), estimated glomerular filtration rate (eGFR), cardiac biomarkers, concomitant drugs in use, echocardiography parameters, and closure size were collected from the electronic medical records on admission.

### Statistical Analyses

This trial was established to compare the impact of different DOAC agents on platelet activation biomarkers following LAAC. Based on the results of a previous clinical study (Asmarats et al., 2020), this study supposed a difference in platelet activation biomarker variations between the different anticoagulants in a prespecified analysis. Therefore, to preserve a one-sided type I error of 5% and adequate power, a sample size of 92 participants was selected to demonstrate a non-inferiority of different DOAC agents on platelet biomarker changes. Assuming the subsequent losses to follow-up to be 15%, a whole study population with 105 patients was required.

The descriptive statistical results of continuous variables were expressed as mean ± standard deviations (SDs), and those of discrete variables were expressed as percentages. One-way independent Student’s *t*-test was used to compare the differences of continuous variables between the two groups of patients, and chi-squared test was performed to compare the correlation of categorical variables.

Differences between the baseline and 3-month follow-up values of the platelet activation biomarkers were studied by using the paired Student’s *t*-test. Platelet activation biomarkers were compared between patients, with and without DRT. A multivariate logistic model was applied to investigate the impact of the CHA_2_DS_2_-VASc score, potential risk factors, LAA size, platelet activation biomarkers, TRAP-induced PA, and anticoagulants on DRT. Linear regression was used to assess the correlation of platelet activation biomarkers with TRAP. D-dimer level was used as a marker for thrombosis after LAAC mainly due to its wide use and recognition as a predictable marker for clinical practices as compared to other markers.

Odds ratios (ORs) with two-sided 95% confidence intervals (CIs) were calculated for the risk factors of a composite end point. Results are presented as ORs along with 95%CI. Statistical analyses were conducted using SPSS^®^ (IBM SPSS Statistics 22.0) and Prism 5 (GrandPad Software). A *p* value of less than 0.05 was considered to be statistically significant.

## Results

### Patient Characteristics

**FIGURE 1:**
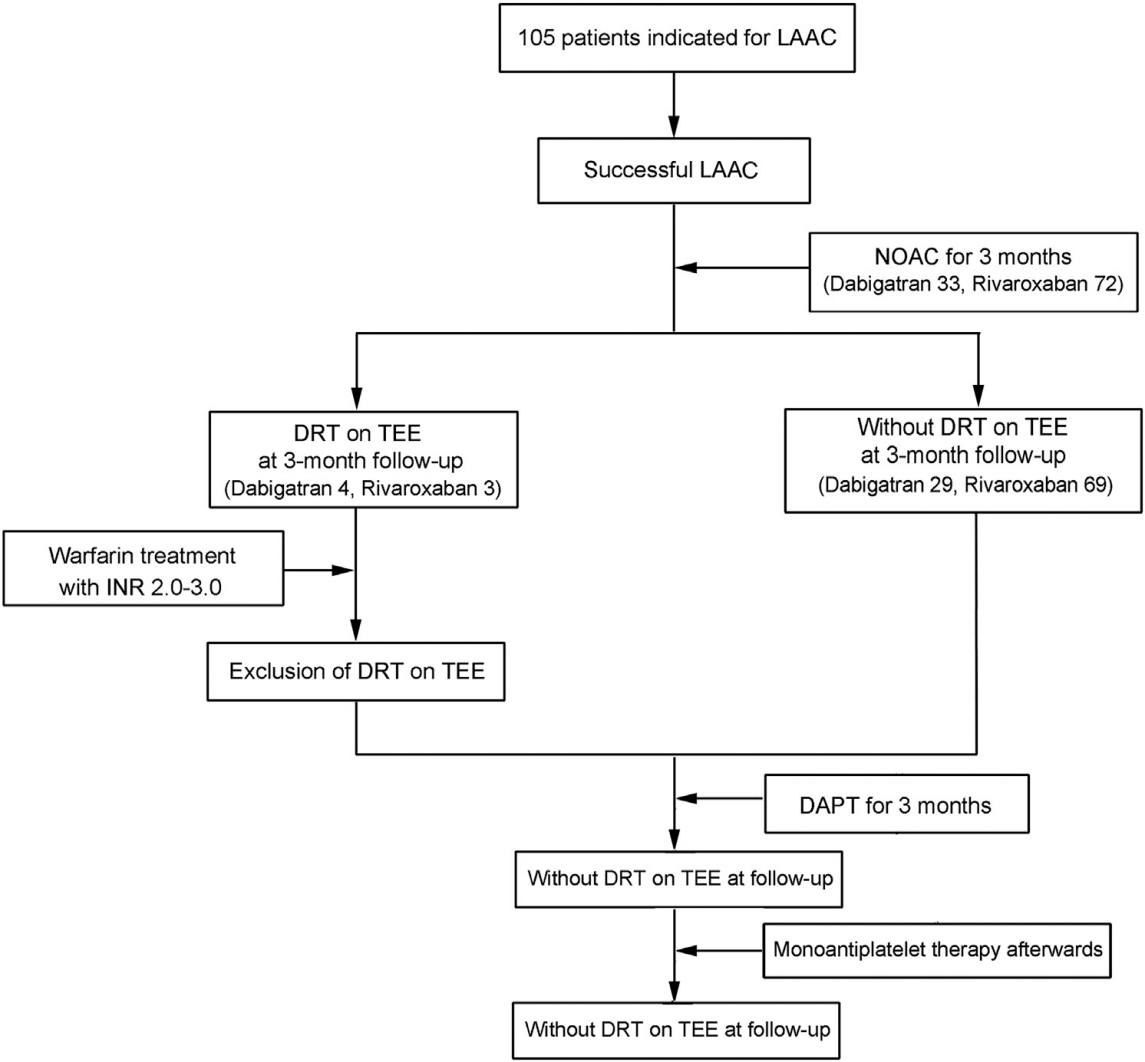
Diagrammatic presentation of the sample size and progression in this study. NOAC, novel oral anticoagulants; DAPT, dual antiplatelet therapy; LAAC, left atrial appendage closure.

During the study inclusion period, a total of 105 consecutive patients with atrial fibrillation underwent percutaneous LAAC operation successfully and completed 3-month anticoagulation with DOAC agents. Among the enrolled patients, 33 (31.4%) patients received dabigatran and 72 (68.6%) received rivaroxaban. After operation, seven patients had developed DRT as confirmed by TEE (four for dabigatran and three for rivaroxaban) and were switched to warfarin anticoagulation with a targeting INR of 2–3 until thrombosis resolution. The study design and progression are summarized in Figure 1.

Table 1 summarizes the demographic and baseline characteristics of the patients. The two groups were well-matched with respect to age, gender, and comorbidity, including diabetes mellitus, hypertension, stroke, and coronary artery disease (*p* > 0.05). Regarding the LAAC procedures, no significant difference was found between the groups in terms of the echocardiography parameters and closure size (*p* > 0.05). The proportion of patients with high thromboembolic risk (CHA_2_DS_2_-VASc score >4) was 55.6% for rivaroxaban and 70.8% for dabigatran. Patients with high bleeding risk (HAS-BLED score >3) accounted for 93.8 and 100% in the rivaroxaban and dabigatran groups, respectively.

**TABLE 1:** Baseline characteristics of the study population.

Baseline Characteristics	Rivaroxaban (*n* = 72)	Dabigatran (*n* = 33)	*p* Value
Age (years), mean (SD)	69.04 ± 8.96	66.17 ± 12.35	0.218
Gender (male), *n* (%)	34 (47.2%)	20 (60.6%)	0.326
Smoking, *n* (%)	8 (11.1%)	3 (9.1%)	0.696
Alcohol, *n* (%)	4 (5.5%)	2 (6.1%)	0.876
Complication			
Hypertension, *n* (%)	44 (61.7%)	17 (51.5%)	0.305
Hyperlipid, *n* (%)	5 (6.9%)	2 (6.1%)	0.576
Diabetes, *n* (%)	15 (21.0%)	4 (12.5%)	0.352
Stroke, *n* (%)	28.4%	25.0%	0.744
Imaging			
LA (mm), mean (SD)	49.04 ± 7.612	46.29 ± 6.083	0.109
LVDd (mm), mean (SD)	47.78 ± 5.812	45.54 ± 5.291	0.494
LVDs (mm), mean (SD)	31.02 ± 5.126	29.13 ± 3.687	0.594
LVEF (%), mean (SD)	63.48 ± 6.762	65.08 ± 4.662	0.281
LAA (mm), mean (SD)	25.31 ± 3.611	24.63 ± 3.132	0.404
Closure size (mm), mean (SD)	28.98 ± 3.420	28.13 ± 3.301	0.284
Laboratory tests			
eGFR (mL/(min·1.73 m^2^)), mean (SD)	75.25 ± 14.71	80.71 ± 21.16	0.154
Hb (g/L), mean (SD)	133.43 ± 15.57	130.50 ± 19.30	0.446
Hct (%), mean (SD)	39.79 ± 4.15	39.18 ± 5.26	0.559
PLT (100*10^9^/L), mean (SD)	185.51 ± 57.40	205.96 ± 53.31	0.122
ALT (U/L), mean (SD)	23.33 ± 19.72	24.04 ± 14.16	0.869
AST (U/L), mean (SD)	24.16 ± 13.63	24.25 ± 7.87	0.976
APTT (s), mean (SD)	29.17 ± 5.14	28.83 ± 6.14	0.783
PT (s), mean (SD)	14.68 ± 5.44	13.48 ± 4.24	0.320
CHA_2_DS_2_-VASc, mean (SD)	3.94 ± 1.34	4.33 ± 1.47	0.218
CHA_2_DS_2_-VASc ≥ 2, *n* (%)	100%	100%	-
CHA_2_DS_2_-VASc ≥ 3, *n* (%)	90.1%	91.7%	0.821
CHA_2_DS_2_-VASc ≥ 4, *n* (%)	55.6%	70.8%	0.181
HAS-BLED, mean (SD)	3.69 ± 0.89	3.88 ± 0.85	0.372
HAS-BLED ≥ 3, *n* (%)	93.8%	100%	0.212
Co-medication			
Statin, *n* (%)	30 (41.7%)	14 (42.4%)	0.942
Anti-hypertension, *n* (%)	57 (79.2%)	22 (66.7%)	0.168
Anti-arrythmic agent, *n* (%)	33 (45.8%)	17 (51.5%)	0.588

The data are shown as mean (SD) or %. SD, standard deviation; LA, left atrial; LVDd, left ventricular end diastolic dimension; LVDs, left ventricular end systolic dimension; LVEF, left ventricular ejection fraction; LAA, left atrial appendage; eGFR, estimated glomerular filtration rate; Hb, hemoglobin; Hct, hematocrit; PLT, platelet; ALT, alanine aminotransferase; AST, aspartate transaminase; APTT, activated partial thromboplastin time; PT, prothrombin time; thrombosis and bleeding risk are represented as CHA_2_DS_2_-VASc and HAS-BLED score, respectively.

### Thrombin Receptor–Activating Peptide–Induced Platelet Aggregation

**FIGURE 2:**
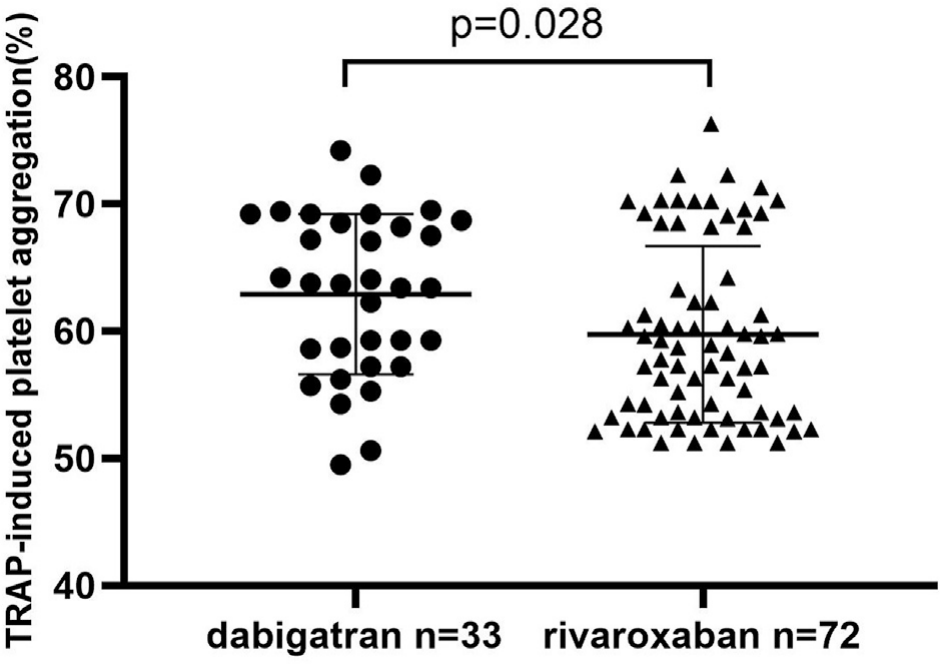
TRAP-induced platelet aggregation: a comparison between dabigatran and rivaroxaban.

In order to investigate whether DOAC agents have an additional clinical effect on the inhibition of PA, TRAP-induced PA was analyzed in patients who had completed 3 months of dabigatran or rivaroxaban use. As shown in Figure 2, the TRAP-induced PA was higher in the dabigatran group than in the rivaroxaban group (62.9 ± 6.3% vs 59.7 ± 6.9%, *p* = 0.028*).

**FIGURE 3:**
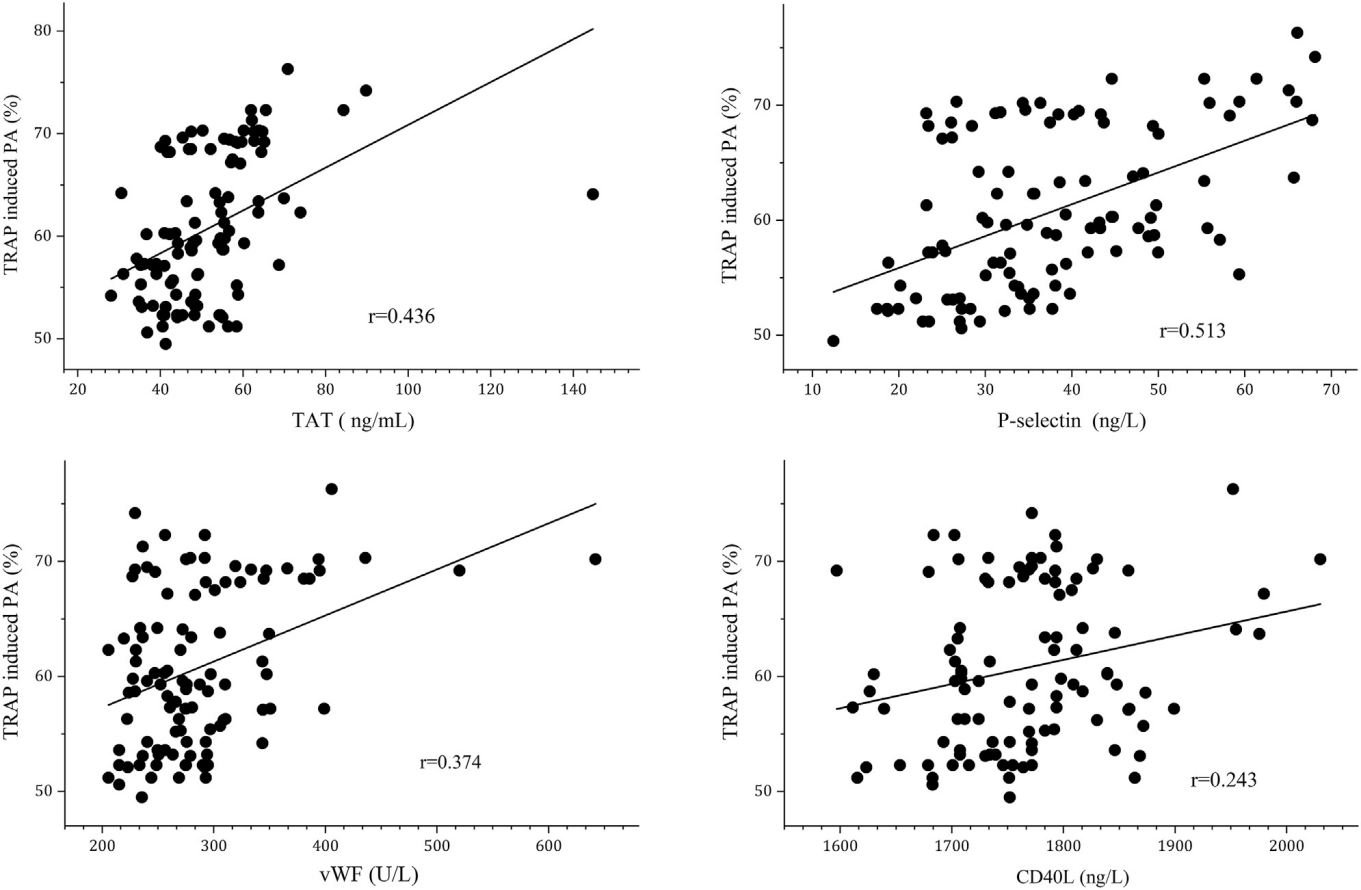
Correlation between TRAP-induced PA and platelet activation biomarkers.

We subsequently performed correlation analysis was performed to further differentiate the relationship of TRAP-induced PA and platelet activation biomarkers. It showed that plasma TAT, P-selectin, and vWF levels were positively correlated with TRAP-induced PA (*r* = 0.436, *r* = 0.513 and *r* = 0.374, respectively) after 3 months of anticoagulation, as shown in Figure 3.

### Plasma Platelet Activation Biomarkers Variation After Direct Oral Anticoagulation Treatment

Regarding the blood samples collected during DOAC anticoagulation on the last day of the 3-month follow-up for the 105 patients were evaluated, and a potential enhanced platelet activation biomarker variations in terms of TAT, P-selectin, and vWF were observed in the dabigatran group as compared to rivaroxaban group, as shown in Table 2. Meanwhile, no significant difference was found with variation of the CD40L plasma levels between the two groups.

**TABLE 2:** Comparison of platelet activation biomarker variation.

Platelet activation biomarkers	Dabigatran (*n* = 33)	Rivaroxaban (*n* = 72)	*p* Value
ΔTAT	13.52 ± 4.17	2.04 ± 1.88	0.001^*^
ΔP-selectin	14.40 ± 2.16	4.79 ± 1.42	0.031^*^
ΔvWF	63.82 ± 11.95	19.57 ± 6.27	0.007^*^
ΔCD40L	30.88 ± 13.83	18.02 ± 9.23	0.543

Intraindividual plasma platelet activation biomarkers variation followed by 3-month post-anticoagulation with dabigatran or rivaroxaban. Δ referred to variation of platelet activation biomarkers; TAT, thrombin–antithrombin complex; vWF, von Willebrand disease.

### Device-Related Thrombosis and Platelet Activation

TEE imaging was available for all enrolled patients, and DRT was recorded throughout the follow-ups. At the end of the study, there were 3 (4.2%) and 4 (12.1%) patients experiencing DRT in the rivaroxaban and dabigatran groups (OR = 0.315, 95%CI: 0.066–1.489, *p* = 0.129), respectively. And the above seven participants experienced no ischemic events, including stroke, myocardiac infarction, and venous thromboembolism during the follow-ups.

**FIGURE 4:**
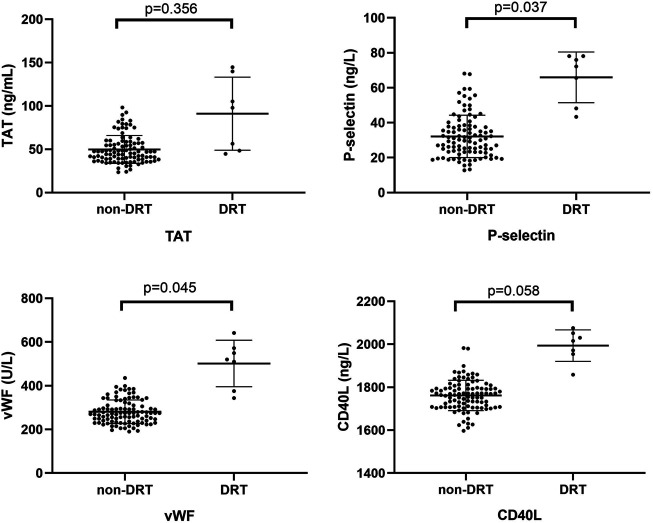
Comparison of platelet activation biomarkers between the DRT and non-DRT groups. P-selectin and vWF plasma levels were significantly higher in the DRT compared with the non-DRT group (*p* < 0.05). This was not seen in the TAT and CD40L groups (*p* > 0.05).

There was a significantly higher expression of P-selection and vWF in the DTR group as compared with the non-DRT group (65.28 ± 13.93 ng/L vs 32.14 ± 12.11 ng/L, *p* = 0.037; 501.92 ± 106.48 U/L vs 280.98 ± 54.10 U/L, *p* = 0.045; respectively), as shown in Figure 4. Meanwhile, no significant difference was found between the DRT and non-DRT groups with respect to changes in TAT and CD40L levels (91.05 ± 42.18 g/ml vs 49.76 ± 16.07 g/ml, *p* = 0.356 and 1993.87 ± 73.31 ng/L vs 1761.70 ± 70.43 ng/L, *p* = 0.058).

Multivariate logistic regression was performed to identify the independent associations of DRT with CHA_2_DS_2_-VASc thrombosis risk scores, age, LAA size, plasma platelet activation biomarkers, TRAP-induced PA, and the use of dabigatran. In the multivariate analysis, the use of dabigatran (*p* = 0.022; OR = 4.366, 95%CI: 0.434–10.839) was a risk factor for DRT following LAAC (Table 3).

**TABLE 3:** Multivariate logistic regression of the DRT occurrence with clinical risk factors.

Variables	SE	*p* Value	OR	95%CI
Age (years)	0.303	0.184	0.669	0.370–1.211
Use of dabigatran (%)	3.152	0.022*	4.366	0.434–10.839
CHA_2_DS_2_-VASc score	3.012	0.202	4.273	1.043–17.543
LAA (mm)	0.528	0.836	0.896	0.318–2.524
TAT (g/mL)	0.321	0.195	1.517	0.808–2.847
P-selectin (ng/L)	0.806	0.173	1.125	0.950–1.132
vWF (U/L)	0.155	0.138	1.259	0.929–1.706
CD40L (ng/L)	0.011	0.612	0.995	0.947–1.016
TRAP-induced platelet aggregation	0.947	0.149	0.255	0.040–1.631

OR indicates odds ratio; 95%CI indicates 95% confidence interval. Multivariate logistic regression included TAT, P-selectin, vWF, and CD40L as covariates along with age, anticoagulation regimen, LAA size, and TRAP-induced platelet aggregation. CHA_2_DS_2_-VASc score was also included as a covariate. * represented with P < 0.05.

### Predictors of D-Dimer > 0.5 mg/ml After 3-month Anticoagulation

**FIGURE 5:**
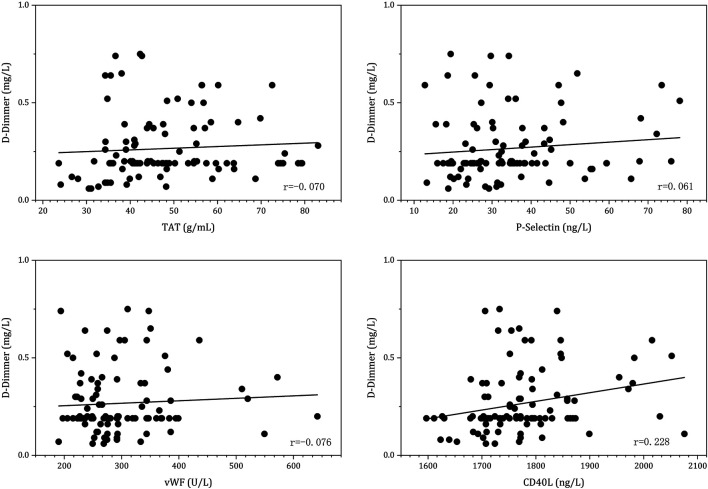
Linear regression between D-dimer and platelet activation biomarkers under 3-month post-anticoagulation with DOAC agents.

Linear regression was conducted to evaluate the relationship between the coagulation status and platelet activation biomarkers after 3 months of anticoagulation with DOAC agents. The results are displayed in Figure 5. The results indicated that CD40L was positively correlated with D-dimer values (*r* = 0.228, *p* = 0.042).

Among the 105 patients enrolled, 16.2% (17/105) had a D-dimer level of more than 0.5 mg/ml, indicating a higher thrombotic risk after 3 months of DOAC post-LAAC. Multivariate regression analysis revealed that CHA_2_DS_2_-VASc score (*p* = 0.016; OR = 2.076, 95%CI: 1.145–3.76) and CD40L (OR = 1.015; *p* = 0.021, 95%CI: 1.002–1.028) were independent predictors of increased D-dimer level, as shown in Table 4.

**TABLE 4:** Multivariate logistic regression of D-dimer > 0.5 mg/ml after 3-month anticoagulation following LAAC.

Variables	SE	*p* Value	OR	95%CI
Age (years)	0.949	0.156	0.261	0.041–1.674
Use of dabigatran (%)	1.062	0.199	3.910	0.488–31.347
CHA_2_DS_2_-VASc score	0.304	0.016^*^	2.076	1.145–3.765
DM	0.976	0.119	0.218	0.032–1.477
Hypertension	0.435	0.237	1.919	0.652–5.644
Stroke history	0.759	0.483	1.703	0.385–7.534
CKD	0.934	0.304	2.612	0.419–16.278
Thrombosis history	2.747	0.830	0.555	0.003–120.887
TAT (g/mL)	0.028	0.240	0.967	0.915–1.023
P-selectin (ng/L)	0.034	0.722	1.012	0.948–1.081
vWF (U/L)	0.007	0.204	0.991	0.978–1.005
CD40L (ng/L)	0.006	0.021^*^	1.015	1.002–1.028
TRAP-induced platelet aggregation	0.053	0.643	1.025	0.923–1.138

CKD, Chronic kidney disease; * represented with P < 0.05.

### Clinical Outcomes

During the 3-month anticoagulation follow-up, no substantial differences were observed between the two groups in terms of incidence of systemic thromboembolism, including stroke and cardiac embolism, for LAAC patients. The bleeding occurrence was not remarkably different between the two groups. There were no significant differences between the two groups with respect to the levels of coagulation parameters as APTT, PT, and PLT (Table 5).

**TABLE 5:** Clinical outcome assessments of the study population.

	Dabigatran (*N* = 33)	Rivaroxaban (*N* = 72)	*p* Value
Stroke (%)	0	1.4%	1.000
Cardiac embolism (%)	3.0%	2.8%	1.000
Bleeding (%)	6.1%	4 (5.6)	1.000
APTT > 31s (%)	18 (54.5)	32 (44.4)	0.336
PT > 13s (%)	17 (51.5)	35 (48.6)	0.782
PLT < 100 × 10^9^ (%)	2 (6.1)	3 (4.2)	0.648

APTT, activated partial thromboplastin time; PLT, platelet count; PT, prothrombin time.

## Discussion

To the best of the authors' knowledge, this study is the first study to investigate the impact of dabigatran and rivaroxaban on platelet reactivity in patients who underwent percutaneous LAAC. The major findings of this study are as follows: 1) platelet activation biomarkers (TAT, P-selectin, and vWF) tended to increase, and PA was activated by dabigatran; 2) dabigatran was associated with higher DRT rate mediated by increasing P-selectin and vWF levels; 3) CHA_2_DS_2_-VASc thrombosis score and CD40L levels may be important predictors of thrombosis after 3-month anticoagulation following LAAC. Based on this study, rivaroxaban has been shown to be superior in preventing periprocedural DRT; therefore, it is suggested that uninterrupted rivaroxaban should be used as the choice of anticoagulant in patients undergoing occluder implantations or LAAC.

Many studies have provided potential clinical benefits of post-anticoagulation with DOAC agents (mainly rivaroxaban) following LAAC (Enomoto et al., 2017; Duthoit et al., 2020). But the optimal anticoagulant remains uncertain due to the lack of clinical trials comparing clinical efficacy and safety between different DOAC agents for LAAC. Previous studies reported that dabigatran was associated with a higher rate of myocardial ischemia due to enhanced platelet reactivity mediated by increasing platelet thrombin receptor expression (Achilles et al., 2017; Sokol et al., 2018), but whether other DOAC agents behave in the same way is unclear. Traditionally, TRAP-induced PA is regarded as a valuable method to reflect platelet inhibition. Consistent with the study by Olivier et al. (2016), the results of this study indicate that dabigatran is associated with higher TRAP-induced PA as compared with rivaroxaban (62.9% vs 59.7%, *p* = 0.028). Furthermore, it was observed that increasing platelet activation biomarkers indicated enhanced platelet activity with dabigatran. One potential explanation of dabigatran-induced PA might be through the enhancement of thrombin receptor density on thrombocytes, which leads to the formation of thrombosis (Achilles et al., 2016). The activation of thrombin receptor peptides was shown to be involved in thrombin-induced platelet activation in previous studies. Olivier et al. (2016) and Vinholt et al. (2017) also found that TRAP-induced PA was enhanced in patients with cardiovascular diseases who were receiving dabigatran, and it was associated with an increased expression of thrombin receptors on the surface of platelets (Vinholt et al., 2017). On the other hand, rivaroxaban could contribute to attenuation of platelet activity and aggregation by inhibiting coagulation factor Xa, which is a factor that increases platelet activity likely *via* Protease Activated Receptor-1 (PAR-1) (Anand et al., 2018; Petzold et al., 2020).

The results of this study on PA triggered by agonists are consistent with previously published data showing the neutrality of rivaroxaban and dabigatran with regard to PA (Jourdi et al., 2019). It was previously suggested that dabigatran might enhance PAR-1 density on platelets (Achilles et al., 2017). DOAC inhibits thrombin generation and thus might indirectly delay PA. Thrombin generated on the platelet surface acts as a potent activator of PAR-1 and PAR-4, resulting in PA (Rigano et al., 2018). Dabigatran increased the lag-time and decreased the velocity more significantly than did rivaroxaban. This difference probably results from the more potent delay of thrombin generation induced by dabigatran following coagulation activation.

Increasing the risk of thromboembolism using direct thrombin inhibitors such as dabigatran has been widely discussed (Chan et al.. 2020), whereas there have been few literatures reporting the changes of platelet activation biomarkers with the use of dabigatran following LAAC. The results of this study showed significant elevation of platelet markers with dabigatran use as compared with that of rivaroxaban. The plasma TAT level has been described as an important coagulation parameter to reflect the prothrombotic state (Castillo et al., 2019). Elevated plasma TAT concentrations were observed with dabigatran, whereas no significant change was observed for rivaroxaban. This is consistent with previous findings that dabigatran might inhibit negative feedback of thrombin, thrombomodulin, and protein C formation, which leads to thrombin generation (Vinholt et al., 2017). _V_WF is involved in many pathological processes including the formation of thrombosis and inflammation (Kawecki et al., 2017). In this study, dabigatran increased the expression of vWF, which led to inflammation, endothelial dysfunction, and oxidative stress. It also increased angiogenesis and the expression of cell adhesion molecules (Feinauer et al., 2021). At the molecular level, PA was attributed to the interaction between P-selectin on platelets and P-selectin glycoprotein ligand-1 on leukocytes (Xia et al., 2002). The results of this study show that there is an increasing expression of plasma P-selectin after long-term dabigatran treatment. According to previous experiments, P-selectin expression could facilitate the binding of platelet and leukocyte by the activation of molecular cascades (Feinauer et al., 2021). This phenomenon was not found under rivaroxaban treatment. Another adhesive protein on the platelet surface, CD40L, also played an important role in the interaction of platelets and leukocytes (Li et al., 2008). However, no significant changes with the CD40L level were observed with either dabigatran or rivaroxaban in this study.

A previous study indicated that it would take about 30–90 days for occluders to be partially or completely endothelialized (Lindner et al., 2021). The implanted devices are more thrombogenic during the first few weeks after LAAC operation mainly due to their exposure to the circulating blood (Moons et al., 2002). The results indicated that DRT might be driven by increasing platelet activation biomarkers following LAAC, and it is possibly related to platelet reactivity and fibrin deposition on the surface of the device. This finding is consistent with preclinical research that showed fibrin deposition on device surfaces during the early periods after LAAC (Subramanian et al., 2018). The enhanced platelet reactivity might reflect the incomplete neo-endocardial coverage of the closures in some cases. In this study, it was noticed that DRT was associated with higher expressions of P-selectin and vWF. This finding agreed with a previous hemostatic marker study in patients who underwent LAAC, and this increased platelet activity might also explain some DRT cases after LAAC operation (Achilles et al., 2017). Compared with dabigatran, there were less incidences of DRT at 90 days with the use of rivaroxaban, indicating that early rivaroxaban use was largely protective. Meanwhile, the marked increase of DRT incidences in the dabigatran group suggests that it might enhance platelet reactivity in some LAAC cases. In this study, the follow-up TEE imaging displayed a higher ratio of DRT with dabigatran, with the need for adjustment of the anticoagulant.

D-dimer which is commonly used as the fibrinolysis marker in clinical practice could represent thrombin generation indirectly (Sadanaga et al., 2010). Increasing D-dimer levels that might reflect a hypercoagulative state and be indicative of vascular injuries with the implanted device have been used to predict the risk of stroke and mortality in previous studies after the administration of DOAC agents in patients with NVAF (Yanagisawa et al., 2019). It has been shown that the D-dimer level increases along with the plasma CD40L level in patients with LAAC (*r* = 0.228). Elevated D-dimer levels may be characteristic of patients with atrial fibrillation who are at the highest risk of thromboembolism, and these patients may benefit the most from anticoagulation therapy. A previous study demonstrated that D-dimer and the marker for ongoing platelet activation, sCD40L, but not P-selectin, were significantly increased in patients with detected LA thrombus. This study shows similar findings in that the levels of D-dimers and CD40L may be the markers predicting LA thrombus. The probable explanation could be that CD40L enhances platelet activation, aggregation, and adhesion on the endothelial cells, which are important factors for atherothrombosis formation (Ellingsen et al., 2019). Above 95% of CD40L circulating in the plasma originate from the platelets (André P Fau - Nannizzi-Alaimo et al., 2002). The increasing plasma CD40L concentration among patients with LAAC who have received 3-month anticoagulation indicate ongoing platelet activation. These results show that CD40L concentration may be used as a predictor for the hypercoagulative state in patients post-LAAC. Factor Xa inhibitors, such as rivaroxaban, have been proven to moderately reduce platelet activity.

## Limitation

There were many limitations in this study. First, the observational nature of the study limits the ability to draw an accurate conclusion. In the future, large prospective and randomized controlled trials are needed to evaluate the clinical effect and adverse drug reactions of DOAC agents for patients post-LAAC. Second, the very low rate of DRT renders such a study impractical. A larger sample size will be needed in further studies. Third, the clinical significance of DRT was not evaluated in the patient population mainly for the low event rate of DRT and thromboembolic events. Finally, only the occurrence of DRT was collected at first follow-up TEE after device implantation in this study. It is certainly true that DRT can occur early after device implantation, which might have an impact on the time to DRT.

## Conclusion

DRT has been acknowledged to have a strong correlation with the risks of postoperative stroke and systematic embolic events. Overall, the data from this study provide an important addition to existing literatures comparing the efficacy and safety between DOAC agents for post-procedural anticoagulation in patients undergoing LAAC. The findings of this study may help guide the choice of anticoagulant in clinical settings. In order to avoid peri-procedural DRT, it may be better to use an anti-Xa inhibitor, such as rivaroxaban, versus a direct thrombin inhibitor, such as dabigatran, for post-procedural anticoagulation in patients post-LAAC. Furthermore, it has been found that platelet activation is involved in many pathological processes, including coagulation, and facilitates the development of DRT. Therefore, a strategy of uninterrupted Factor Xa inhibitor administration could be used routinely in patients during and after implantation of occluders, as they have been shown to be safe in preventing peri-procedural DRT. However, these results need to be further confirmed in future clinical studies.

## Data Availability

The original contributions presented in the study are included in the article/Supplementary Material, and further inquiries can be directed to the corresponding authors.
